# Supplementary material for: Tracking objects outside the line of sight using 2D intensity images
Source: Sci Rep. 2016 Aug 31;6:32491. doi: 10.1038/srep32491 (PMC5006175; doi:10.1038/srep32491)
Supplement: Supplementary Information [file srep32491-s1.pdf]

Tracking objects outside the line of sight using 2D intensity images

Jonathan Klein[2,1], Christoph Peters[1], Jaime Martín[1], Martin Laurenzis[2], and Matthias B. Hullin[1,*]

[1]University of Bonn, Institute of Computer Science II, Bonn, Germany
[2]French-German Research Institute Saint-Louis, France
[*] hullin@cs.uni-bonn.de

Supplementary Video

This video shows two real-time tracking sessions (Session 1: translation only; Session 2: translation and rotation) using the described setup. A live view of the hidden scene is shown next to the output from the tracking software. The average reconstruction rate during these tracking sessions was 10.2 frames per second (limited by the maximum capture rate of our camera-laser setup) for Session 1, and 3.7 frames per second (limited by computation) for Session 2. The 2-dimensional car model was represented by 502 surfels; the total compute time required for a single tracking step was 72.9 ms for translation only, and 226.1 ms for translation and rotation.
